# Highly Sensitive and Selective Fluorescent Probes for Cu(II) Detection Based on Calix[4]arene-Oxacyclophane Architectures

**DOI:** 10.3390/molecules25102456

**Published:** 2020-05-25

**Authors:** Alexandra I. Costa, Patrícia D. Barata, Carina B. Fialho, José V. Prata

**Affiliations:** 1 Departamento de Engenharia Química, Instituto Superior de Engenharia de Lisboa, Instituto Politécnico de Lisboa, R. Conselheiro Emídio Navarro, 1, 1959-007 Lisboa, Portugal; acosta@deq.isel.ipl.pt (A.I.C.); pbarata@deq.isel.ipl.pt (P.D.B.); carina.fialho@tecnico.ulisboa.pt (C.B.F.); 2 Centro de Química-Vila Real, Universidade de Trás-os-Montes e Alto Douro, 5001-801 Vila Real, Portugal

**Keywords:** calix[4]arene, copper, supramolecular, inclusion complex, fluorescence, sensor, density functional theory

## Abstract

A new topological design of fluorescent probes for sensing copper ion is disclosed. The calix[4]arene-oxacyclophane (Calix-OCP) receptor, either wired-in-series in arylene-*alt*-ethynylene conjugated polymers or standing alone as a sole molecular probe, display a remarkable affinity and selectivity for Cu(II). The unique recognition properties of Calix-OCP system toward copper cation stem from its pre-organised cyclic array of *O*-ligands at the calixarene narrow rim, which is kept in a conformational rigid arrangement by a tethered oxacyclophane sub-unit. The magnitude of the binding constants (*K*_a_ = 5.30 − 8.52 × 10^4^ M^−1^) and the free energy changes for the inclusion complexation (−Δ*G* = 27.0 − 28.1 kJmol^−1^), retrieved from fluorimetric titration experiments, revealed a high sensitivity of Calix-OCP architectures for Cu(II) species. Formation of supramolecular inclusion complexes was evidenced from UV-Vis spectroscopy. The new Calix-OCP-conjugated polymers (polymers **4** and **5**), synthesized in good yields by Sonogashira–Hagihara methodologies, exhibit high fluorescence quantum yields (*Φ*_F_ = 0.59 − 0.65). Density functional theory (DFT) calculations were used to support the experimental findings. The fluorescence on–off behaviour of the sensing systems is tentatively explained by a photoinduced electron transfer mechanism.

## 1. Introduction

Molecular fluorescent systems for signalling supramolecular interactions have been in use for more than forty years [1,2]. When aimed for ion detection, the system usually comprises either a fluorogenic unit covalently linked through some sort of a spacer to an ion receptor or an integrated fluorophore-ion recognition site assembly [1,2,3]. The photophysical changes that occur upon cation binding, which are the basis for signalling the binding event, may have different origins. The main underlying mechanisms are based on photoinduced electron transfer (PET), photoinduced charge transfer (PCT), excimer-formation/disappearance and Förster resonance energy transfer (FRET) processes [4]. The electronic and stereochemical characteristics of the recognition unit and the fluorophore, as well as the type of metal ion, dictate the actual mechanism. With transition metal ions, examples covering all the above-mentioned processes are known [4].

Calixarenes are cyclic oligomers widely investigated due to their ability to recognize and differentiate neutral and ionic guests through the formation of inclusion complexes [5,6]. Various topologies of fluorescent sensors for metal cation recognition based on calixarene-derived molecular receptors have been developed [7]. Of these, those targeting transition metal ions are of particular relevance for the present study. In general, sensors are built by attaching the fluorogenic unit(s) to the narrow rim of the calixarene receptor. The most used signalling elements belong to naphthyl, anthracenyl, pyrenyl, dansyl, quinolinyl, and benzimidazolyl groups [4,7]. On the receptor side, calixarenes having cone or 1,3-alternate conformations are usually employed [4,7].

Copper(II), lead(II) and mercury(II) ions are ubiquitous in nature, either associated to important physiological processes [8] or representing environmental hazards and health issues [9,10,11,12,13], making their sensitive and selective detection highly desirable. The literature regarding the use of calixarene-based fluorescence sensors for recognition of the above transition metal cations have been recently reviewed and will not be further detailed here [14].

Both off–on (fluorescence enhancement) [15,16,17] and on–off (fluorescence quenching) [18,19,20] responses have been used for signalling the binding events with copper ion, using the above described assemblies of calixarene receptors and fluorophore units, usually with high sensitivities.

**Chart 1:**
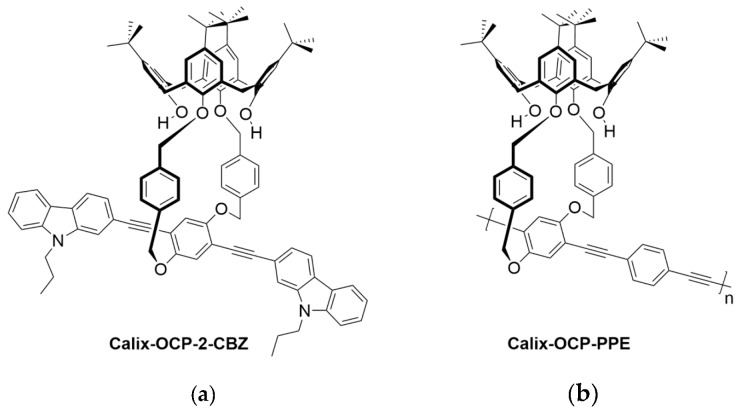
Chemical structures of **Calix-OCP-2-CBZ** (**a**) [28] and **Calix-OCP-PPE** (**b**) [26].

**Scheme 1:**
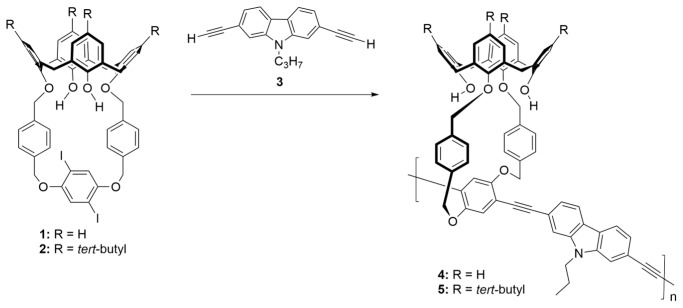
Cross-coupling of calix[4]arene diiodo derivatives **1** and **2** with 2,7-diethynyl-9-propyl-9*H*-carbazole (**3**) using PdCl_2_(PPh_3_)_2_/CuI catalytic system in toluene/NEt_3_ at 35 °C for 24–48 h.

The above reports stimulated our interest to screen the ability of a series of fluorescent calix[4]arene platforms we have synthesized in the past, mostly designed as probes for nitroaromatic explosives [21,22], nitroaliphatic explosives and taggants [23,24], nitroanilines [25,26], and proteins [27], as sensors of transition metal ions, specifically Cu(II), Pb(II) and Hg(II). A particular family of calixarene derivatives having an oxacyclophane (OCP) unit tethered to the narrow rim of a calixarene unit [25], either as single-molecules (**Calix-OCP-2-CBZ**; Chart 1a) [28] or integrated in a polymer (**Calix-OCP-PPE**; Chart 1b) [26] was envisaged as having potential for transition metal ions recognition. This stems from their well-defined cone conformations, which may offer suitable pre-organised ion binding sites at the Calix-OCP skeleton. The enhanced rigidity of the calix[4]arene structure provided by the OCP sub-unit, which concomitantly defines the size of the recognition centre, may potentiate the selectivity of binding to metal ions as a function of their size and charge. Therefore, they were selected for the current study. In addition, two new conjugated polymers differing in the substitution at the *para* position of aryl rings (with and without *tert*-butyl groups) were constructed from the above calixarene scaffolds, having in their chains phenylene-*alt*-ethynylene-*alt*-2,7-carbazolylenes as repeating units (***p*-H-Calix-OCP-PAE-2,7-CBZ** (**4**) and ***p-tert*-butyl-Calix-OCP-PAE-2,7-CBZ** (**5**); Scheme 1). It is expected that the good donor ability of the carbazole moieties could enhance the sensing capabilities of the whole assembly in comparison to similar calixarene architectures possessing phenylene-*alt*-ethynylene conjugated units along the polymer chain [*cf*. **Calix-OCP-PPE**].

Screening of this pool of potential supramolecular probes will possibly allow a series of interesting observations and useful conclusions to be reached. One will be what entity is responsible for recognition of the metal ion (site specificity). The other will focus on any sensitive enhancement of sensing behaviour promoted by conjugated polymer chains (two types in appreciation) in comparison to the corresponding sole molecular probe. A third will be to what extent the electronic/stereochemical nature of the conjugated polymer backbone influences the signal transduction of the recognition event. The last will result from the evaluation of the impact of the substitution pattern at the upper rim of the calixarene unit on the sensitivity and selectivity of the sensor system.

## 2. Results and Discussion

### 2.1. Synthesis of Polymeric Fluorescent Probes

The new fluorescent polymers (**4** and **5**) were synthesized by a Pd-catalysed Sonogashira–Hagihara methodology. Cross-coupling of oxacyclophane tethered calix[4]arenes (Calix-OCPs) **1** [29] and **2** [25] (Scheme 1) with 2,7-diethynyl-9-propyl-9*H*-carbazole (2,7-CBZ) monomer **3** [30] furnished the desired polymers. Polymerisations were carried out in toluene/NEt_3_ at 35 °C for several hours (24–48 h), using PdCl_2_(PPh_3_)_2_/CuI as the catalytic system (Scheme 1). The polymers were isolated as yellow solids in good yields (58–66%). The polymers are moderately soluble in CH_2_Cl_2_, CHCl_3_, and THF, and only sparingly soluble in CH_3_CN.

Gel permeation chromatography (GPC) was used to estimate the polymers’ molecular weights (see GPC traces in Figure S1; Supplementary Materials). GPC analysis of the isolated polymers showed the presence of less than 1% of monomers in their composition. Data for polymers **4** and **5** are summarised in Table 1.

Formation of a reddish gel-like insoluble material was noted during the isolation of polymers, being this fraction higher for polymer **4**. This may explain the lower yield of its isolated soluble fraction, and also the lower DP attained in its isolated fraction (DP = 5).

### 2.2. Structural Characterization of Polymers

FTIR and ^1^H NMR analyses were used to structurally characterise the new materials. One characteristic FTIR feature of the synthesized polymers is the disappearance of terminal ethynylic C≡C-H stretching vibrations characteristic of 2,7-CBZ monomer unit [30] and the concomitant presence of internal alkyne frequencies at 2202 cm^−1^ (**4**) and 2204 cm^−1^ (**5**). This indicates that the majority of the polymer chains are end-terminated by iodine. Other relevant vibrational frequencies may be found in the Experimental section. Analysis of FTIR spectra of the insoluble fractions resulting from the polymerisations leading to polymers **4** and **5** showed they are similar to those obtained from the corresponding soluble fractions. The insoluble fractions should then represent polymers with higher degrees of polymerisation, which render them insoluble.

The somewhat limited solubility of polymers **4** and **5** in CDCl_3_, CD_2_Cl_2_, or DMSO, in proper concentrations for NMR experiments, allied to the usual broadening of signals in complex polymer matrices, prevent the acquisition of good quality ^1^H NMR spectra. Nonetheless, the spectral assignments that were tentatively made (see Experimental section) fully confirm the proposed structures. In particular, the cone conformation of the calixarene units in both polymers was substantiated by the existence of a set of characteristic resonances for the diastereotopic protons of bridged methylene groups in the calixarene skeleton. For polymer **5** the equatorial protons present signals at 3.22 ppm (2H, d, *J* = 13.6 Hz), 3.33 ppm (2H, d, *J* = 13.2 Hz), and at 4.25–4.50 ppm (4H, two unresolved doublets) for the axial protons. The corresponding resonances in **4** appear at 3.25-3.45 ppm (4H, two types of unresolved doublets) and 4.32–4.42 ppm (4H, two unresolved doublets), respectively. The two types of signals for equatorial protons present in **4** and the pattern of resonances for the diastereotopic benzylic protons (4.90–5.62 ppm) is an indication of a much less conformationally fixed calixarene structure. NMR spectra of **4** and **5** are displayed in Figures S2 and S3.

### 2.3. Photophysical Properties

**Figure 1:**
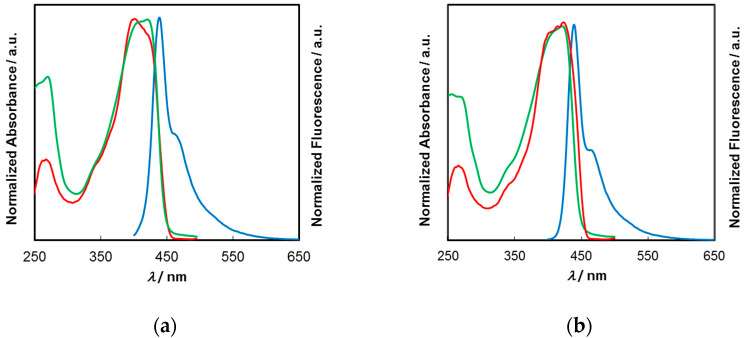
Absorption (green line), excitation (red line, monitored at emission maxima) and emission (blue line) spectra (*λ*_exc_ = 380 nm, CHCl_3_) of polymer **4** (**a**) and polymer **5** (**b**).

The ground–state absorption, excitation and emission spectra of the polymers **4** and **5** are depicted in Figure 1.

Polymers **4** and **5** have similar absorption profiles, both peaking around 420 nm at their absorption maxima. A large molar absorptivity is observed for **5**. Note the similarity of the absorption maxima of polymers despite their different degrees of polymerisation; this means that the mean conjugation length of the polymer chain reaches a plateau within at least around five repeating units. The optical HOMO-LUMO energy gaps (*E*_g_), calculated from the low energy onset of the absorption bands of the polymers (*E*_g(**4**)_ = 2.87 eV; *E*_g(**5**)_ = 2.86 eV) are in line with the above observations.

Polymers **4** (*Φ*_F_ = 0.65) and **5** (*Φ*_F_ = 0.59) are highly fluorescent in CHCl_3_ solution. Their emission profiles are again very similar, presenting 0–0 transitions at 431 nm for **4** and a slightly redshift emission for polymer **5** at 439 nm, followed by discernible 0-1 vibronic progressions at 458 and 467 nm, respectively. In each case, relatively short Stokes shifts were found. The main photophysical properties of **4** and **5** are gathered in Table 2.

### 2.4. Inclusion Complexes and Sensing of Metal Cations

The sensing ability of our integrated receptor–fluorophore systems toward metal ions are expected to closely follow their aptness for supramolecular complex formation. Their sensorial activity was assessed through fluorimetric titrations. The experiments were carried out by titrating diluted CH_3_CN solutions of fluorescent polymers (mostly at 5.0 × 10^−6^ M) with metal cations as perchlorates salts. The photostability of polymers **4** and **5** in CH_3_CN was first evaluated under the conditions used in the titration experiments (*λ*_exc_ = 380 nm, right angle illumination). No photodegradation occurs in any of the synthesized polymers up to 1 h of continuous irradiation.

The Stern–Volmer expression

*F*_0_/*F* = 1 + *K*_SV_ [Q] (1)

derived for static quenching (with *K*_SV_ = *K*_a_) [31], or any of its modified forms, such as the reciprocal fitting (Benesi–Hildebrand or Lineweaver–Burk) or double logarithmic plots (Scatchard), are widely used to calculate the association constant in binding events. However, they may furnish erroneous results. In Equation (1), *K*_SV_ is the static Stern–Volmer constant (also denoted as *K*_S_) and [Q] is the free quencher concentration. As several authors have noticed [32,33,34], there are two main reasons why these formalisms may fail to produce accurate results. One is to consider that the host–guest complex once formed is completely non-fluorescent and the other is the assumption that the concentration of added guest during a titration experiment (and later used in Stern–Volmer plots from which *K*_SV_ is extracted) is identical to that of the free guest (the one featuring in Equation (1). Deviations from the true *K*_a_ value are more significant in systems with large binding affinities, in which the concentration of free guest is considerably lower than that of added guest. Following the above reasoning, all the association constants presented in this work were calculated by a non-linear fitting approach (see below) because it is a more accurate fitting method, and no a priori assumptions need to be made.

The extent of complexation in fluorimetric assays was quantified as follows. The fluorescence intensity of the unbound host (H, calixarene species) in diluted solutions is expressed through the equation

*F* = *ε*_F_[H] = (ln10)*Φ*_F_*I*_0_*ε* l [H] (2)

where *ε*_F_ is the molar fluorescence intensity [35]. Changes in fluorescence quantum yield (*Φ*_F_) and/or in the molar absorptivity (*ε*) after exposure to a given analyte, while keeping the exciting intensity (*I*_0_) and path length (l) constant, may lead to observable changes in the fluorescence intensity, which constitutes the basis of fluorescence titration. The association constant (*K*_a_) for the formation of a supramolecular complex (HG) between the host and the metal guest (G), from the 1:1 equilibria H + G ⇄ HG, is described by

*K*_a_ = [HG]/[H][G] (3)

By combining Equations (2) and (3), the quadratic equation

Δ*F*^2^ − Δ*ε*_F_([H]_0_ + [G]_0_ + 1/*K*_a_) Δ*F +* Δ*ε*_F_^2^[H]_0_[G]_0_ = 0 (4)

may be derived [36]. The association constant of the binding event may then be calculated by solving Equation (4) for Δ*F* (Equation (5)):

Δ*F* = ½{Δ*ε*_F_([H]_0_ + [G]_0_ + 1/*K*_a_) − [Δ*ε*_F_^2^([H]_0_ + [G]_0_ + 1/*K*_a_)^2^ − 4Δ*ε*_F_^2^[H]_0_[G]_0_]^1/2^} (5)

where Δ*F* and Δ*ε*_F_ are the changes in fluorescence intensity and molar fluorescence intensity of the host upon complexation with metal ions, *K*_a_ is the association constant, and [H]_0_ and [G]_0_ denote the initial concentrations of the host and the guest, respectively. Using a non-linear regression analysis, the association constant of the complex can be retrieved using Δ*F*, [G]_0_ and [H]_0_ as input parameters. This equation holds for a 1:1 host-to-guest equilibrium and considers a neglectable dynamic quenching component for the system under study.

Before applying this model, it was verified that the reduction of emission intensity is only due to host-guest interactions and, therefore, artefacts such as hetero inner-filter effects (h-IFEs) by metal salts were absent. Indeed, the metal salts used in this work do not exhibit absorption of radiation at the excitation (380 nm) or emission wavelengths (400–470 nm) of the fluorophores; thus, no h-IFEs could occur in these systems.

**Figure 2:**
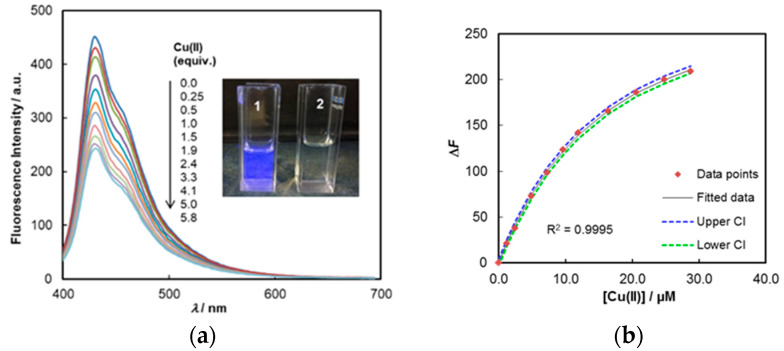
(**a**) Emission spectra of polymer **5** (5.0 × 10^−6^ M in CH_3_CN) upon addition of increasing amounts (0.25 − 5.8 equivalent) of Cu(ClO_4_)_2_ (*λ*_exc_ = 380 nm). Inset: photo of polymer **5** fluorescence under UV irradiation (366 nm) before (**1**) and after (**2**) Cu(ClO_4_)_2_ addition (5 equivalent); (**b**) Binding isotherm for the fluorimetric titration of **5** with Cu(II) with fitted curve and confidence intervals (see text).

Polymer **5** was assayed in first place. As depicted in Figure 2a, the emission of **5** is strongly quenched upon contact with increasing amounts of Cu(II), reaching a 44% reduction of the initial fluorescence intensity with the addition of 5 equivalent of copper ion.

**Figure 3:**
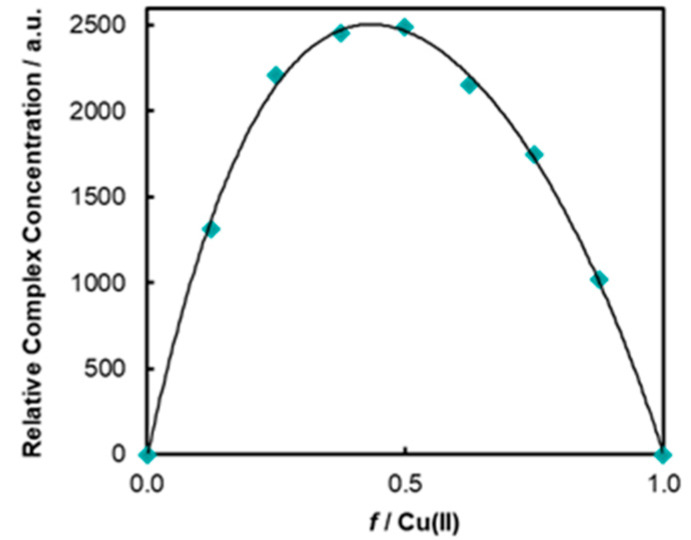
Job plot for complex formation between host **5** and Cu(II) in CH_3_CN (at constant 1.0 × 10^−5^ M total concentration) as obtained from changes in fluorescence (*λ*_exc_ = 380 nm).

Titration data and curve-fitting plots for the supramolecular titration of polymer **5** with Cu(II) are shown in Figure 2b. Regression analysis was performed by the Solver function in Microsoft Excel [37] using the non-linear generalised reduced gradient (GRG) algorithm, from which a *K*_a_ = (8.52 ± 0.82) × 10^4^ M^−1^ was derived. The excellent goodness of fit (R^2^ = 0.9995) confirms the 1:1 stoichiometry of the supramolecular complex. The degree of uncertainty was estimated from the upper and lower confidence intervals (CI) for fitted data (Figure 2b), calculated from the standard error of residuals and the critical *t*-value [37,38]. The stoichiometry of the complex was also assessed by the continuous variation method (Job’s method). By plotting the concentration of the host–guest complex ([HG]) against the mole fraction (*f*) of the guest, the stoichiometry of the complex can be found for *f* corresponding to the maximum of [HG]. The relative complex concentration was calculated from [HG] = (*F*_0_ − *F*)/*F*_0_ × [H], where (*F*_0_ − *F*)/*F*_0_ is the relative fluorescence intensities and [H] the concentration of pure **5**. The Job plot (Figure 3) exhibits a maximum at a mole fraction of 0.5 indicating the formation of a complex with n:n stoichiometry (1:1, 2:2, or higher) between **5** and Cu(II). Full evidence for a 1:1 complex was obtained after application of a described method (Table S1) [39].

A previously designed conjugated polymer (**Calix-OCP-PPE** (**6**); DP = 35) [26], possessing the same recognition unit (*tert*-butyl-calix[4]arene-oxacyclophane) as **5** but holding instead phenyleneethynylene entities along the polymer chain (Chart 1b), was next evaluated for its recognition ability. The titration data (Figure S4a) were fitted by the non-linear procedure yielding an association constant of (5.30 ± 1.27) × 10^4^ M^−1^ (Figure S4b). As with polymer **5**, a very high sensitivity was observed.

**Chart 2:**
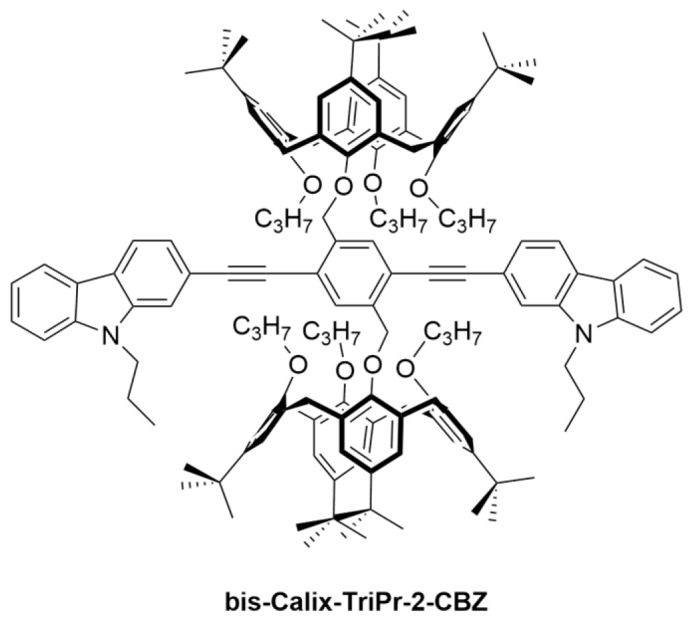
Chemical structure of **bis-Calix-TriPr-2-CBZ** [23].

In order to get further insights about the role of conjugated polymer backbones in polymers **5** and **6** on the hypothetical amplification of the binding event, likely occurring at the narrow rim of the calixarene sub-unit (see below), the molecular receptor **Calix-OCP-2-CBZ [28]** (Chart 1a), having in common with the former polymers the calixarene-OCP framework, was investigated. Following similar fitting procedures, it was found that this sole molecule also displayed a high binding affinity for copper ion (*K*_a_ = (6.62 ± 1.92) × 10^4^ M^−1^), albeit with a higher statistical uncertainty when compared to **5** (Figure S5a,b). On the other hand, a bis-calix[4]arene derivative, **bis-Calix-TriPr-2-CBZ [23]** (Chart 2), having the same fluorophore element (bis-2-CBZ-phenyleneethynylene) but lacking the calix-oxacyclophane architecture did not show any responsiveness to Cu(II) (Figure S6). It results obvious that the fluorophore unit is not able by itself to give any direct response to the presence of the analyte.

From the above findings, one may formulate that for a strong binding event to occur, and subsequently a high sensitivity in detection could be observed, it is absolutely essential the existence of a conformationally rigid cyclic array of *O*-ligands at the narrow rim of calixarene derivative functioning as the cation receptor. Calixarene-OCP structures provide such a binding site in which the four oxygen ligands are very well positioned, and possess the appropriate inter-atomic distances between them that will incidentally allow an effective interaction with incoming cations. Of course, the proper match between the size of the ligand pocket and the size of the cation will dictate the relative success of the binding, and also any observable selectivity. This is what apparently occurs in the case of polymers **5** and **6**, and **Calix-OCP-2-CBZ**, where the copper ion fits well into the host cavity.

It has been shown by single-crystal X-ray diffraction that in polynuclear clusters formed from *p*-*tert*-butyl-calix[4]arenetetraol and Fe(III) [40] or Cu(II) [41], the four narrow rim oxygen atoms bind to either of the metal ions in a square-planar geometry. Density functional theory (DFT) calculations on *p*-H-calix[4]arenes containing appended coumarins [42] also predicted binding through the phenolic oxygens to Cu(II) and Fe(III) cations.

**Figure 4:**
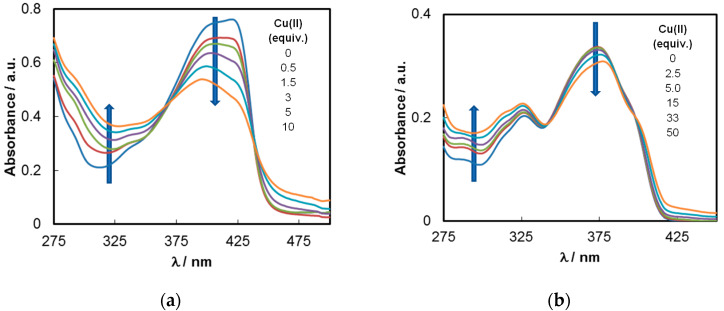
Absorption spectra of (**a**) polymer **5** (8.2 × 10^−5^ M in CH_3_CN:CHCl_3_, 1:1) and (**b**) **Calix-OCP-2-CBZ** (5.0 × 10^−6^ M in CH_3_CN) upon addition of increasing amounts of Cu(ClO_4_)_2_.

Support for the occurrence of a ground-state complex between polymer **5** and Cu(II) was brought about by UV-Vis spectroscopic titrations. As shown in Figure 4a, a stepwise increment of the amount of copper ion (up to 10 equivalent) causes appreciable changes in the absorption profile. In the range 275–350 nm, an increase in absorbance is assignable to a ligand-to-metal charge transfer (LMCT) absorption band, whereas an intense hypochromic effect appears within the π-π* transition region of the conjugated polymer system. Two clear isosbestic points appear at 370 and 439 nm denoting the establishment of the binding equilibrium. Similar features appear in the titration of **Calix-OCP-2-CBZ** with Cu(II), also showing the LMCT band and a less pronounced chromic effect on the region of phenyleneethynylene-2-CBZ chromophore (Figure 4b). The two isosbestic points are located at 340 and 398 nm.

To further elucidate the above interactions, a Calix-OCP-Cu(II) model was constructed. It is known that Cu(II) forms coordination compounds with different geometries (tetra- to hexa-coordinate) [43]. In the present case, and for the analysis of any conformational changes occurring in the calixarene structure upon binding, a square planar tetra-coordinate geometry for Cu(II) was considered for modelling purposes. Even if solvated forms of the complex truly exist, for example with CH_3_CN bound at one or two apical positions of Cu(II) leading to putative square pyramidal or octahedral geometries, respectively, this should not interfere with the main conclusions to be drawn from the model.

**Figure 5:**
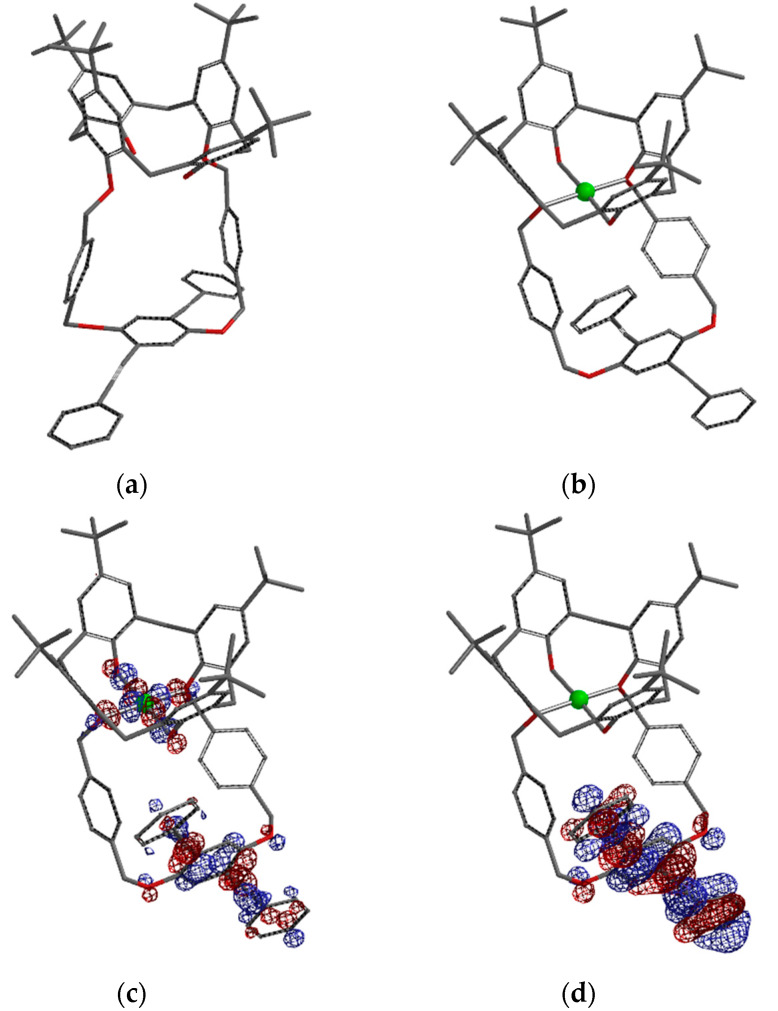
Energy and geometry optimised structures of Calix-OCP (**a**) and Calix-OCP-Cu(II) (**b**) models (side views). β-HOMO (**c**) and α-HOMO (**d**) molecular orbitals mapped on the structure of the complex (side views). DFT calculations run at the B3LYP/6-31G (**d**) level of theory in vacuum [44]. Hydrogens omitted for clarity. Colour codes for elements: red = oxygen, grey = carbon, green = copper.

The energy and geometry of the complex were optimised by DFT calculations at B3LYP/6-31G(d) level of theory [44] (see details in Materials and Methods section). On complexation with Cu(II) ion, the structure of the host, having in its unbound state a pinched cone conformation (*C*_2v_ symmetry) in the calixarene unit (Figure 5a), adopts an almost perfect cone arrangement (*C*_4v_ symmetry) (Figure 5b). Besides, the attached oxacyclophane ring changes its pristine conformation dramatically in order to better accommodate the copper ion.

The average distance between the distal oxygen ligands (d_O1-O3_ = 3.222 Å and d_O2-O4_ = 4.953 Å) at the narrow rim of the free Calix-OCP-model in the pinched cone conformation is 4.088 Å, meaning a mean distance of 2.044 Å to their centroid. On the other hand, the sum of the cation ionic radius of Cu(II) (r_ion_ = 0.57 Å) [45] and the van der Waals oxygen atom radius (r_vdW_ = 1.52 Å) [44] is 2.09 Å. This shows that the binding pocket of Calix-OCP is very well suited for receiving the copper ion, after proper conformational rearrangement. Inspection of the model’s structure showed that the copper complex adopts a square planar configuration with a very low degree of distortion (dihedral angle (θ) between the planes formed by O-Cu-O is 177°), having O-Cu bond lengths of O(H)-Cu = 2.001 Å, O(H)-Cu = 2.019 Å, O(CH_2_Ar)-Cu = 2.002 Å and O(CH_2_Ar)-Cu = 1.990 Å, yielding a mean distance between the *O*-ligands and the central Cu(II) in the complex equal to 2.003 Å.

The strong binding of Cu(II) with Calix-OCP systems that has been experimentally observed is supported by the high binding energy (Δ*E* = − 1616.6 kJ/mol) obtained by DFT calculations for the model compound with Cu(II) in gas-phase. The coordinative bonding of *O*-ligands to Cu(II) is reflected on the resulting ground-state Mulliken charge of the metal ion after complexation, which is reduced from +2.000, in its unbound state, to +0.812.

Upon complexation, the copper guest is held in close proximity (<6 Å) to the transduction centres located either at the polymers backbone (polymers **5** and **6**) or at the bis-2-CBZ-phenyleneethynylene units (**Calix-OCP-2-CBZ**). Given the electron acceptor characteristic of the complexed Cu(II), where the β-LUMO contains an appreciable orbital coefficient in the copper ion (d_x_^2^_−y_^2^ orbital) (Figure 5c), and the electron donor nature of the conjugated phenyleneethynylene-aryl systems (where the α-HOMO is located; Figure 5d), it is conceivable that, after complexation and light excitation, a rapid photoinduced electron transfer (PET) ensues from the singlet excited state (^1^SOMO*) of the donor to the β-LUMO, followed by a non-radiative back electron transfer to the fluorophore, leading to the observed fluorescence quenching (for a schematic illustration, see Figure S7).

The absence of any shift in the maxima of excitation or emission spectra of polymer **5**-Cu(II) complex (Figure S8), or in its ground-state absorption spectra (Figure 4), reinforces the idea that the observed fluorescence quenching occurs by a PET mechanism and not by a photoinduced charge transfer (PCT) mechanism [4].

From the above sensing results a few conclusions may be drawn. First, the structures containing phenyleneethynylene-CBZ elements (polymer **5** and **Calix-OCP-2-CBZ**) are better reporters for signalizing the formation of the complex, than polymer **6**. This is because the systems bearing carbazole units, which possess electron rich N atoms, raise the HOMO and LUMO energies [24]. The HOMO, on excitation, lead to a higher-energy ^1^SOMO*, which, in turn, result in more exergonic electron transfers. Secondly, the higher response of polymer **5** in comparison to **Calix-OCP-2-CBZ** (*ca*. 30% of *K*_a_ enhancement) can be attributed to the ability of **5** to amplify the transduction signal through energy migration along the conjugated polymer backbone [46,47].

The sensorial ability of polymers **5** and **6** and **Calix-OCP-2-CBZ** toward Cu(II) is quite remarkable particularly when compared to other already reported excellent sensors. For non-polymeric probes, a calix[4]arene containing two pyrenyl amine groups showed a *K*_sv_ of 1.49 × 10^4^ M^−1^ [48], while a series of calixarene receptors with appended dansyl groups display association constants ranging from 1.45 × 10^3^ − 6.31 × 10^4^ M^−1^ [49]. Pyrenyl-isoxazolo-tethered calix[4]arenes also display significant responses to Cu(II) with *K*_a_ in the range 6.00 × 10^3^ − 4.71 × 10^4^ M^−1^ [50]. When considering conjugated polymers, either fluorene-*alt*-phenylene and phenyleneethynylene-*alt*-thienyleneethynylene type polymers, containing attached, respectively, *N*-cyclohexyl carbamates (*K*_sv_ = 1.10 × 10^4^ M^−1^) [51] and tolylterpyridyl (*K*_sv_ = 2.01 × 10^5^ M^−1^) [52] groups as recognition elements, have been used for Cu(II) detection.

Having established the noteworthy sensitivity of polymer **5** toward Cu(II), the next step dealt with the selectivity of the recognition. For this, its fluorescence response toward two other transition metal ions, Pb(II) and Hg(II), was evaluated. Notably, titration experiments with these ions do not reveal any significant or measurable response (Figures S9 and S10). A similar behaviour was followed by **Calix-OCP-2-CBZ**, whereas no affinity for the lead ion was displayed (Figure S11). These high selectivities, in addition to their outstanding sensitivity already revealed, turns polymer **5** and **Calix-OCP-2-CBZ** very efficient platforms for Cu(II) detection. It is worth mentioning that in several previous works using calix[4]arene-based ionophores, particularly those relying in dansyl groups [49] sensitivities for Cu(II) and Pb(II) are of the same order of magnitude, whereas for Hg(II) the affinity was in general even higher, overall making the attained selectivity for Cu(II) in these systems rather poor. In other works [50], a good selectivity was achieved for Cu(II) in comparison, for instance, with Pb(II) or Hg(II), although in a lower sensitivity when compared to polymer **5**.

The reason behind the lack of affinity of polymer **5** toward Pb(II) should be due to a mismatch between the cation size and the available space in the ligand, leading to a weak binding interaction. DFT calculations were used to shed some light on this issue. Pb(II) is a much larger ion (ionic radius = 98 pm; four-coordinate) [45] than Cu(II) (ionic radius = 57 pm; tetra-coordinate) [45]. Figure S12 presents the molecular structure of a putative Calix-OCP-Pb(II) complex (model). As is clear from the model, in order to accommodate the lead ion in a tetra-coordinate complex, a severe distortion of the square planar configuration (θ = 149°) has to occur. This has profound consequences in the binding strength. A measure of this is provided by the predicted Mulliken charge (+1.146) of Pb(II) in the complex. The lower charge transfer from the oxygen ligands to lead cation, in comparison to Cu(II), results in a much less stable complex.

The same observation applies to the absence of measurable binding between polymer **5** and Hg(II), which has a similar ionic radius (ionic radius = 96 pm; four-coordinate) [45] to that of Pb(II).

### 2.5. Effect of Calixarene Structure on Sensing

**Figure 6:**
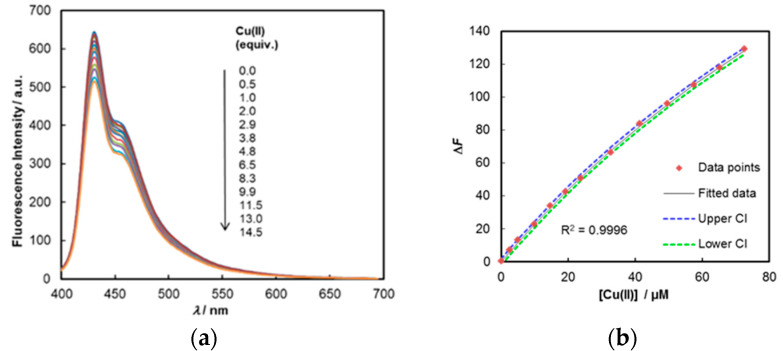
(**a**) Emission spectra of polymer **4** (5.0 × 10^−6^ M in CH_3_CN) upon addition of increasing amounts (up to 14.5 eq.) of Cu(ClO_4_)_2_ (*λ*_exc_ = 380 nm); (**b**) Binding isotherm for the fluorimetric titration of **4** with Cu(II) along with the fitted curve and confidence intervals.

Polymer **4** is the de-*tert*-butylated analogue of **5**. Owing to the absence of *tert*-butyl groups in the calixarene upper rim, the conformational mobility of the calixarene moiety is much higher. This structural lability may have an impact on the binding affinity to metal ions. It is, thus, of interest to compare the behaviour of polymer **4** with polymer **5** in terms of its sensitivity and selectivity. The spectra of **4** upon successive additions of Cu(II) is shown in Figure 6a. After the addition of 5 equivalents of copper ion, the initial emission only drops by 14%. This represents a huge loss of affinity for Cu(II). The curve-fitting plots are depicted in Figure 6b. A *K*_a_ = (5.29 ± 0.78) × 10^3^ M^−1^ was obtained from the regression analysis, which is 16 times lower than that of polymer **5**.

The stoichiometry of the complex is also 1:1, as judged from the goodness of fit (R^2^ = 0.9971) on the non-linear model. The Job plot (Figure S13) exhibits a maximum for the concentration of the complex at a mole fraction of 0.5, again pointing to the formation of a 1:1 supramolecular complex (Table S2).

The association constants and the free energy changes (Δ*G*) for complexation of the various screened hosts with Cu(II) are gathered in Table 3.

From the thermodynamic point of view, and assuming similar entropic losses (e.g., decrease of motion freedom of calixarene-OCP units and rearrangement of solvent molecules around the host and guest upon complexation) for the inclusion complexation of Cu(II) by polymers **4** and **5**, the higher free energy change (Δ*G*) associated to polymer **5** should be driven by the more favourable enthalpy changes, which are a direct result of stronger binding interactions developed in this complex. The same rational applies to polymer **6** and **Calix-OCP-2-CBZ**.

In titrations experiments of polymer **4** with Pb(II), and contrary to the results from the assays with polymer **5** and **Calix-OCP-2-CBZ**, a measurable affinity for this ion was found, with a *K*_a_ = (4.47 ± 1.61) × 10^3^ M^−1^ (Figure S14a,b). The drastic reduction of binding ability of polymer **4** toward Cu(II) and its lower selectivity, for instance in comparison to polymer **5**, can only be traced to the different structure of the calixarene recognition unit. Polymer **4**, being more conformationally mobile than **5**, do not bind so tightly to the metal ion. Indeed, the lack of the *tert*-butyl substituents at the larger rim, allows that the phenyl rings in the cyclic oligomer can adopt a more open geometry at the narrow rim, due to the removal of steric hindrance at the wider rim. This was in part corroborated by modelling studies (same theory as above), from which a lower binding energy (Δ*E* = −1581.9 kJ/mol) was obtained for the complexation of *p*-H-Calix-OCP-Cu(II) model, turning the complex of polymer **5** with Cu(II) more stable by 34.7 kJ/mol.

The same line of thought helps to explain why **4** is also responsive to Pb(II) whereas polymer **5** is almost silent. The conformational flexibility of **4** is apparently sufficient to hold Pb(II) in its cavity, in spite of the larger ionic radius of lead ion.

The above effects are not completely unprecedented for calix[4]arenes in the cone conformation. A survey of the literature showed that an increase of the number of *tert*-butyl groups at the upper rim of thiacalix[4]arenes containing dansyl derivatives decreases the sensing ability of the hosts toward Co(II), Ni(II) and Cd(II) cations [53]. In another report, using dansyl-modified calix[4]arenes, a slight increase in selectivity for Pb(II) over Hg(II) and Fe(III) was registered when the hosts were devoid of *tert*-butyl groups [54].

## 3. Materials and Methods

### 3.1. Instruments and Methods

FTIR were measured on a Bruker Vertex 70 as KBr pellets (transmission mode). ^1^H NMR spectra were collected on Brüker AVANCE II^+^ spectrometers (400 MHz); reported chemical shifts (*δ*/ppm) are internally referenced to CDCl_3_ (^1^H, 7.26 ppm). The splitting parameters for ^1^H NMR are denoted as follows: s (singlet), d (doublet), t (triplet), and m (multiplet).

GPC was performed on a Jasco Liquid Chromatograph system equipped with a Jasco UV Absorption Detector 1575 (selected to 275 nm), using Polymer Standards Service (PSS) styrene-divinylbenzene copolymer (SDV) (PSS SDV) columns (10^3^ and 10^5^ Å) and THF as eluent at 35 °C. Calibration was done with monodisperse polystyrene standards. Number-average (*M*_n_) and weight-average (*M*_w_) molecular weights and polydispersity (*M*_w_/*M*_n_) were calculated from GPC data based on UV response at 270 nm. Polymer concentrations are expressed as per the molecular weight of the repeating unit.

UV-vis spectra were recorded on a Nicolet Evolution 300 or on a Jasco J-815 spectrophotometer using 1-cm quartz cells at 25 °C.

Steady-state fluorescence spectra were acquired on a Perkin Elmer LS45 fluorimeter using a 1-cm quartz cuvette in right angle geometry (RA) at 25 °C in air-equilibrated conditions. Fluorescence quantum yields were measured using 9,10-diphenylanthracene as fluorescence reference standard (*Φ* = 0.72, EtOH, air equilibrated conditions, RA) [55]. The quantum yields were determined by the slope method [56], keeping the optical densities of the sample and reference below 0.05 at the excitation wavelength to prevent inner filter effects.

Titration experiments (UV-Vis and fluorescence) were carried out at a constant concentration of the hosts (5.0 × 10^−6^ M in CH_3_CN, except when noticed otherwise) by adding known amounts of metal perchlorates in CH_3_CN solutions at 25 °C, being the spectra repeatedly acquired after each addition.

### 3.2. Materials

Calixarenes **1** [29] and **2** [25], 2,7-diethynyl-9-propyl-9*H*-carbazole (**3**) [30], **Calix-OCP-PPE** (**6**) [26], **Calix-OCP-2-CBZ** [28], and **bis-Calix-TriPr-2-CBZ** [23] were synthesized according to our reported methods and fully characterized by FTIR, UV-Vis and NMR spectroscopies.

Dichlorobis(triphenylphosphine)palladium (II) (98%, Aldrich), copper(I) iodide (98%, Aldrich), Cu(ClO_4_)_2_ (Acros Organics, 98%), Hg(ClO_4_)_2_.3H_2_O (Alfa Aesar, reagent grade), Pb(ClO_4_)_2_ (Acros Organics, 99%), and 9,10-diphenylanthracene (scintillation grade, Nuclear Enterprises Ltd.) were used as received. Triethylamine (99%, Riedel-de-Haën) was previously dried from CaH_2_ and distilled under N_2_ prior to use. Toluene was previous dried from Na, distilled under N_2_ and stored over Na. All other reagents and solvents were reagent grade and were purified and dried by standard methods. Organic extracts were dried over anhydrous magnesium sulphate.

### 3.3. Polymers’ Synthesis

***p*-H-Calix-OCP-PAE-2,7-CBZ** (**4**): To an argon degassed solution containing 40 mg (40.4 µmol) of **1** in dry toluene (1.6 mL) and freshly distilled NEt_3_ (1.6 mL) were added PdCl_2_(PPh_3_)_2_ (1.98 mg, 2.83 µmol), CuI (0.54 mg, 2.83 µmol) and 11.4 mg (44.4 µmol) of carbazole monomer **3** under argon. The mixture was degassed, the flask sealed, and the contents stirred in a pre-heated bath at 35 °C. The mixture acquired a yellow colour that gradually turned to brown–orangish with turbidity. After 24 h, GPC showed an almost quantitative conversion of **1**. Solvents were removed by evaporation and the residue taken in CH_2_Cl_2_ and washed successively with aqueous solutions of 2% HCl, 0.1M NaHSO_3_, 10% NH_4_SCN and water. The organic extract was dried and evaporated to dryness. The residue was dissolved in a minimum amount of CH_2_Cl_2_ and the polymer precipitated by the addition of MeOH. The product was obtained as a yellow amorphous solid in 58% (23.2 mg). During the CH_2_Cl_2_ dissolution, an insoluble gel-type fraction was obtained. *υ*_max_/cm^−1^ (KBr) 3435, 3060, 3020, 2960, 2925, 2874, 2202, 2137, 1622, 1598, 1559, 1465, 1404, 1371, 1324, 1250, 1214, 1192, 1088, 1016, 846, 805, 753; *λ*_max_/nm (*ε*_max_ × 10^−4^ M^−1^ cm^−1^) 420 (4.18), cutoff at 470 nm; *δ*_H_/ppm (CDCl_3_; 400.130 MHz; Figure S2) 0.92–1.05 (m, 3H, *N*-CH_2_CH_2_CH_3_), 1.85–1.99 (2H, m, *N*-CH_2_CH_2_CH_3_), 3.25–3.45 (4H, m, ArCH_2eq_Ar), 4.18–4.32 (2H, m, *N*-CH_2_CH_2_CH_3_), 4.32–4.42 (4H, m, ArCH_2ax_Ar), 4.90–5.62 (8H, several m, Ar_calix_OCH_2_Ar), 6.61–6.72 (4H, m, Ar_calix_H), 6.73–6.83 (4H, m, Ar_calix_H), 7.01–7.08 (4H, m, Ar_calix_H and 2H, s, ArOH), 7.08–7.13 (2H, s, Ar_phenylene_H), 7.28–7.35 (4H, m, Ar_benzylene-metaArCalix_H, overlapped with CDCl_3_), 7.40–7.48 (2H, m, ArC_(3,6)_H-CBZ), 7.56–7.71 (2H, m, ArC_(1,8)_H-CBZ, and 4H, m, Ar_benzylene-orthoArCalix_H), 7.94–8.05 (2H, m, ArC_(4,5)_H-CBZ).

GPC data (THF solution at 35 °C against polystyrene standards): *M*_n_ = 5276 gmol^−1^; *M*_w_/*M*_n_ = 2.81; degree of polymerisation (DP) = 5 based on *M*_n(GPC)_ (Figure S1).

***p-tert-Butyl*-Calix-OCP-PAE-2,7-CBZ** (**5**). The above procedure was used in the synthesis of **5**, using 40 mg (32.9 µmol) of **2**, 1.3 mL of dry toluene and freshly distilled NEt_3_, 1.6 mg (2.3 µmol) of PdCl_2_(PPh_3_)_2_, 0.43 mg (2.3 µmol) of CuI and 9.3 mg (36.2 µmol) of **3**. After 48 h, polymer **5** was isolated as a yellow amorphous solid in 66% (26.3 mg). During the CH_2_Cl_2_ dissolution, an insoluble gel-type fraction was obtained in vestigial amount. *υ*_max_/cm^−1^ (KBr) 3435, 3050, 2961, 2926, 2903, 2867, 2204, 2138, 1599, 1556, 1483, 1461, 1406, 1363, 1323, 1208, 1124, 1097, 1019, 947, 872, 804; *λ*_max_/nm (*ε*_max_ × 10^−4^ M^−1^ cm^−1^) 422 (4.81), cutoff at 470 nm. *δ*_H_/ppm (CDCl_3_; 400.130 MHz; Figure S3) 0.87 (18H, s, Ar_calixOH_-C(CH_3_)_3_), 0.93–1.06 (3H, m, *N*-CH_2_CH_2_CH_3_), 1.29 (18H, s, Ar_calixOR_-C(CH_3_)_3_), 1.84–2.02 (2H, m, *N*-CH_2_CH_2_CH_3_), 3.22 (2H, d, ArCH_2eq_Ar, *J* = 13.6 Hz), 3.33 (2H, d, ArCH_2eq_Ar, *J* = 13.2), 4.10–4.35 (2H, m, *N*-CH_2_CH_2_CH_3_; partially overlapped with ArCH_2ax_Ar), 4.25–4.50 (4H, m, ArCH_2ax_Ar), 4.85–5.00 (2H, m, Ar_calix_OCH_2_Ar), 5.00–5.15 (2H, m, Ar_calix_OCH_2_Ar), 5.24–5.44 (2H, m, ArOCH_2_Ar), 5.46–5.67 (2H, m, ArOCH_2_Ar), 6.65 (2H, s, ArOH), 6.69 (4H, s, Ar_calixOH_H), 7.04 (4H, s, Ar_calixOR_H), 7.08 (2H, s, Ar_phenylene_H), 7.22–7.39 (4H, m, Ar_benzylene-metaArCalix_H, overlapped with CDCl_3_), 7.40–7.50 (2H, m, ArC_(3,6)_H-CBZ), 7.55–7.75 (2H, m, ArC_(1,8)_H-CBZ and 4H, m, Ar_benzylene-orthoArCalix_H), 7.90–8.12 (2H, m, ArC_(4,5)_H-CBZ).

GPC data (THF solution at 35 °C against polystyrene standards): *M*_n_ = 15,587 gmol^−1^; *M*_w_/*M*_n_ = 4.26; DP = 13 based on *M*_n(GPC)_ (Figure S1).

### 3.4. Computational Methods and Non-linear Regression Analysis

Lowest-energy conformers for Calix-OCP and *p*-H-Calix-OCP models were obtained from conformational searches (Monte Carlo method, MMFF94 force field). They were then subjected to full energy and geometry optimisation by density functional theory (DFT) calculations running on an hybrid model (B3LYP) using 6-31G(d) basis sets, as developed in Spartan’14 [44], using default grids and convergence criteria in vacuum. Calculations involving Pb(II) have used the LANL2DZ pseudopotentials as implemented by default in Spartan’14. The input geometries for the corresponding metal complexes (Calix-OCP-Metal(II) and *p*-H-Calix-OCP-Metal(II)) were prepared by placing the metal(II) at the centroid of the narrow rim of the calixarene structures in a tetra-coordinate geometry. No other ligands (CH_3_CN) or counter-ions (perchlorate ions) were considered in the modelling studies. The lowest-energy conformers were found by the same molecular mechanics method, and then fully optimized by DFT/B3LYP/6-31G(d) level of theory.

The binding energy (Δ*E*) in gas-phase for the complexation has been calculated by the equation Δ*E* = *E*_Complex_ − (*E*_Cu(II)_ + *E*_Calix-OCP_) where *E*_Complex_ is the energy of Calix-OCP-Cu(II) model, *E*_Cu(II)_ the energy of the naked cation, and *E*_Calix-OCP_ the energy of the most stable conformation of the unbound Calix-OCP model.

Non-linear regression analysis was performed by the Solver function in Microsoft Excel [37] using the non-linear generalised reduced gradient (GRG) algorithm, and a convergence criterion for R^2^ < 10^−9^. The critical *t*-value was calculated at a significance level of 95%.

## 4. Conclusions

New fluorescent probes for sensing copper ion in organic media were revealed. Calix[4]arene-oxacyclophane molecular receptors proved to be excellent platforms for the recognition of Cu(II) which, combined with highly responsive fluorophore units as transduction sites, led as a whole to an outstanding sensitivity and selectivity for copper(II). The best signalling entities comprise either a conjugated polymer integrating phenylene-*alt*-ethynylene-*alt*-2,7-carbazolylenes as repeating units (polymer **5**) or an individual molecular species (**Calix-OCP-2-CBZ**) containing a similar structural arrangement of the fluorescent element. The three-dimensionality of the host cavity, providing a conformationally rigid cyclic array of *O*-ligands at the narrow rim of the calixarene fostered by the tethered oxacyclophane sub-unit, was found to be essential for the development of a strong binding between the host and the copper cation. The existence of *tert*-butyl groups at the calixarene wider rim is essential for keeping the host in a more tight conformation, not only allowing much higher sensitive responses but also an enhancement on the selectivity of metal ions recognition based on their sizes.

Experimental evidence from UV-Vis have undoubtedly shown the formation of supramolecular inclusion complexes between the calixarene hosts and Cu(II). A photoinduced electron transfer mechanism between the excited fluorophore and the complexed copper ion was proposed to explain the fluorescence turn-off behaviour of the sensing systems used in this work.

The new sensors here described feature among the best ever reported for copper detection.

Studies are now underway to evaluate the behaviour of Calix-OCP-Cu(II)/Cu(I) inclusion complexes as redox-active centres in biomimetic chemistry.

## Figures and Tables

**Table 1:** Data from the cross-coupling polymerisation of Calix-OCPs (**1** and **2**) with 2,7-CBZ (**3**) ^1^.

Polymer	Yield (%) ^2^	*M*_w_ (g/mol) ^3^	*M*_n_ (g/mol) ^3^	*M*_w_/*M*_n_^3^
**4**	58	14842	5276	2.81
**5**	66	66468	15587	4.26

^1^ Typical conditions: PdCl_2_(Ph_3_P)_2_ (7 mol%), CuI (7 mol%), toluene, NEt_3_, and 2,7-CBZ units (1.1 equivalent), 35 °C, 24–48 h; ^2^ Refer to yields of isolated soluble fractions with less than 1% of **1** or **2** (GPC evaluation); ^3^ Number-average (*M*_n_) and weight-average (*M*_w_) molecular weights and polydispersity (*M*_w_/*M*_n_) were retrieved from by GPC data, using THF as eluent at 35 °C and monodisperse polystyrene standards.

**Table 2:** Photophysical properties of polymers **4** and **5** in CHCl_3_.

Polymer	*λ*_abs max_/nm (log*ε*_max_)	*E*_g_/eV ^1^	*λ*_em max_/nm	Stokes Shift/nm ^2^	*Φ*_F_^3^
**4**	420 (4.621)	2.87	431 ^4^ 458 ^5^	11	0.65
**5**	422 (4.682)	2.86	439 ^4^ 467 ^5^	17	0.59

^1^ Optical energy gaps (*E*_g_) were calculated from the low energy onset of the absorption bands; ^2^ Stokes shifts were calculated from *λ*_F, max_ (0-0) − *λ*_A, max_; ^3^ Fluorescence quantum yields were determined in CHCl_3_ using 9,10-diphenylanthracene as reference in air-equilibrated conditions; ^4^ At the maximum of the first vibronic (0-0) band; ^5^ At the maximum of the second vibronic (0-1) band.

**Table 3:** Association constants (*K*_a_) and free energy changes (Δ*G*) for the inclusion complexation with Cu(II).

Host	*K*_a_/M^−1^	Δ*G*/kJ/mol ^1^
Polymer **4**	5.29 × 10^3^	−21.2
Polymer **5**	8.52 × 10^4^	−28.1
Polymer **6**	5.30 × 10^4^	−27.0
**Calix-OCP-2-CBZ**	6.62 × 10^4^	−27.5

^1^ Δ*G* values were calculated at 25 °C (298 K).

## Supplementary Materials

The following are available online at https://www.mdpi.com/1420-3049/25/10/2456/s1, Figures S1–S14, Tables S1 and S2.
