# Supplementary material for: Highly Sensitive and Selective Fluorescent Probes for Cu(II) Detection Based on Calix[4]arene-Oxacyclophane Architectures
Source: Molecules. 2020 May 25;25(10):2456. doi: 10.3390/molecules25102456 (PMC7287820; doi:10.3390/molecules25102456)
Supplement: Supplementary file 1 [file molecules-25-02456-s001.pdf]

molecules

Article

Highly Sensitive and Selective Fluorescent Probes for Cu(II) Detection Based on Calix[4]arene-Oxacyclophane Architectures

Alexandra I. Costa [1,2], Patrícia D. Barata [1,2], Carina B. Fialho [1,†] and José V. Prata [1,2,*]

1 Departamento de Engenharia Química, Instituto Superior de Engenharia de Lisboa, Instituto Politécnico de Lisboa, R. Conselheiro Emídio Navarro, 1, 1959-007 Lisboa, Portugal; acosta@deq.isel.ipl.pt (A.I.C.); pbarata@deq.isel.ipl.pt (P.D.B.); carina.fialho@tecnico.ulisboa.pt (C.B.F.)
2 Centro de Química-Vila Real, Universidade de Trás-os-Montes e Alto Douro, 5001-801 Vila Real, Portugal
* Correspondence: jvprata@deq.isel.ipl.pt; Tel.: +351-218317172
† Present address: Centro de Química Estrutural, Instituto Superior Técnico, Universidade de Lisboa, Av. Rovisco Pais, 1049-001 Lisboa, Portugal.

Supplementary Materials

Table of Contents

Figure S1. GPC traces of polymers 4 and 5

Figure S2. ^{1}H NMR spectrum of polymer 4

Figure S3. ^{1}H NMR spectrum of polymer 5

Figure S4a. Emission spectra of polymer 6 upon addition of $Cu(ClO_4)_2$

Figure S4b. Binding isotherm for the fluorimetric titration of 6 with Cu(II)

Figure S5a. Emission spectra of Calix-OCP-2-CBZ upon addition of $Cu(ClO_4)_2$

Figure S5b. Binding isotherm for the fluorimetric titration of Calix-OCP-2-CBZ with Cu(II)

Figure S6. Emission spectra of bis-Calix-TriPr-2-CBZ upon addition of $Cu(ClO_4)_2$

Figure S7. Mechanism of fluorescence quenching by PET

Figure S8. Excitation and emission spectra of polymer 5 upon addition of $Cu(ClO_4)_2$

Figure S9. Emission spectra of polymer 5 upon addition of $Pb(ClO_4)_2$

Figure S10. Emission spectra of polymer 5 upon addition of $Hg(ClO_4)_2$

Figure S11. Emission spectra of Calix-OCP-2-CBZ upon addition $Pb(ClO_4)_2$

Figure S12. Optimised structure of Calix-OCP-Pb(II) complex (model)

Figure S13. Job plot for complex formation between polymer 4 and Cu(II)

Figure S14a. Emission spectra of polymer 4 upon addition of $Pb(ClO_4)_2$

Figure S14b. Binding isotherm for the fluorimetric titration of 4 with Pb(II)

Table S1. Experimental values of $\Sigma c/c_{max}$ of the Job plot for polymer 5

Table S2. Experimental values of $\Sigma c/c_{max}$ of the Job plot for polymer 4

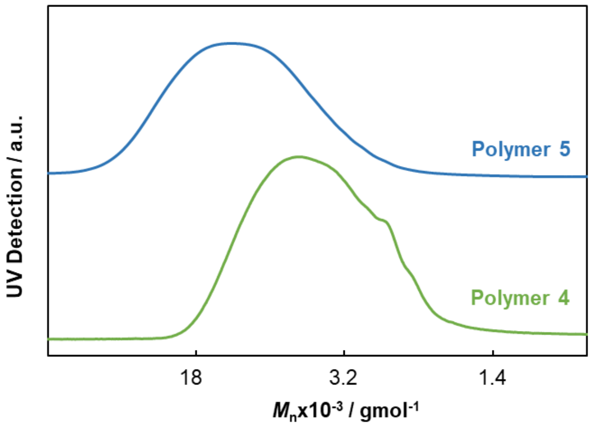

Figure 1. GPC traces of isolated polymers **4** and **5** against monodisperse polystyrene standards (THF as eluent at 35 °C).

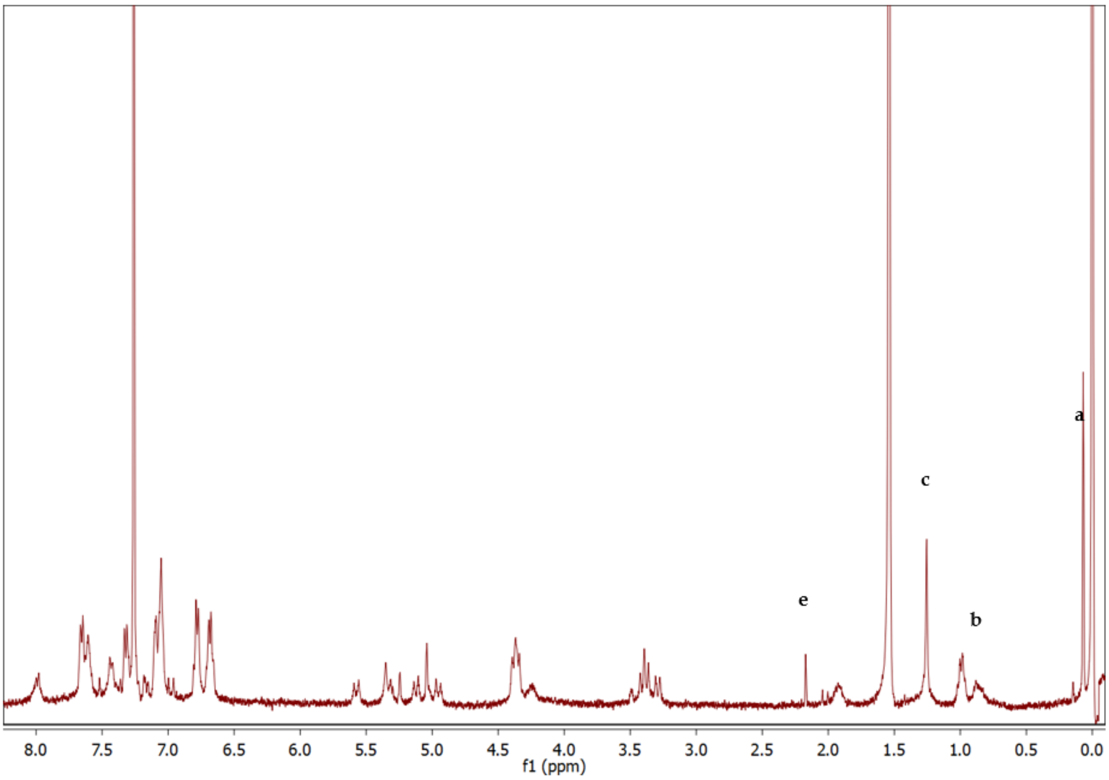

Figure 2. ^{1}H NMR spectrum of polymer **4** in $CDCl_3$ (400 MHz, 25 ºC); [a]silicone grease; [b,c]Apiezon type grease; [d]water; [e]acetone.

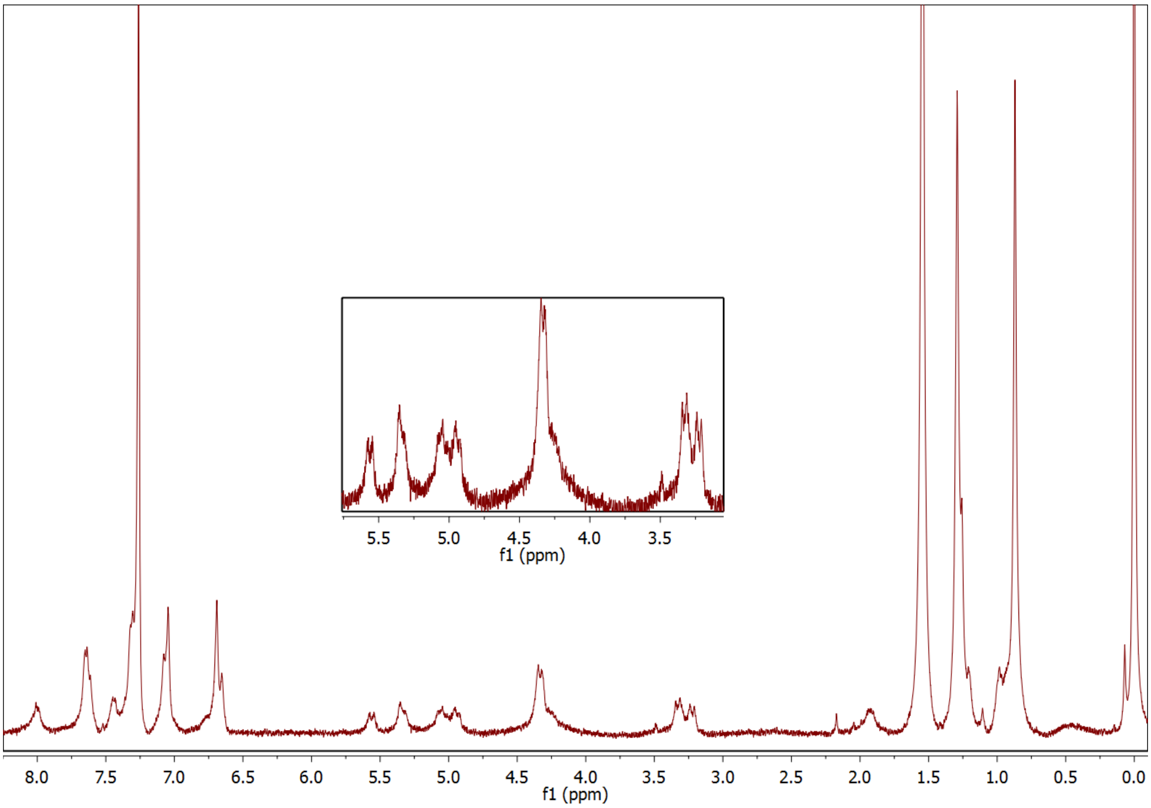

Figure 3. ^{1}H NMR spectrum of polymer **5** in $CDCl_3$ (400 MHz, 25 ºC); [a]silicone grease; [b]Apiezon type grease; [c]water; [d]acetone.

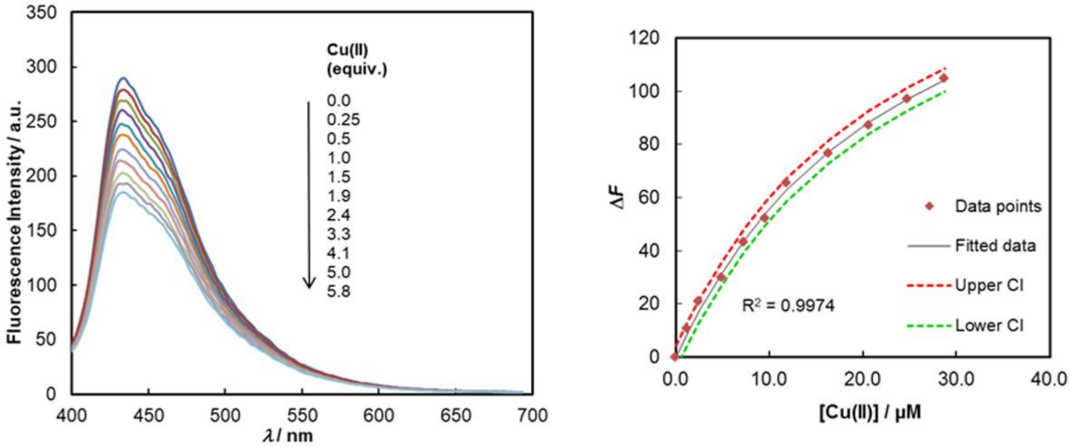

(**a**) (**b**)

Figure 4. (**a**) Emission spectra of polymer **6** (5.0 x 10^{-6} M in CH_3CN) upon addition of increasing amounts (0.25 - 5.8 equiv.) of $Cu(ClO_4)_2$ (λ_{exc} = 380 nm); (**b**) Binding isotherm for the fluorimetric titration of **6** with Cu(II) with fitted curve and confidence intervals.

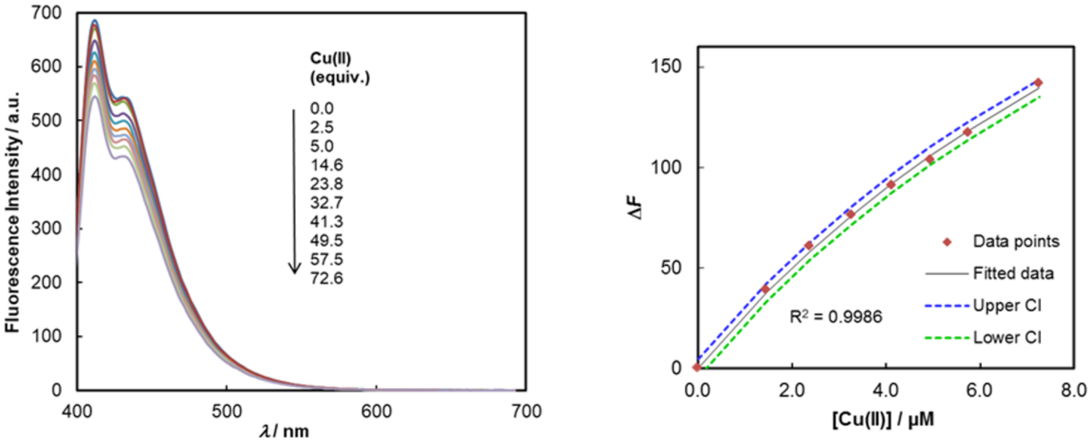

(**a**) (**b**)

Figure 5. (**a**) Emission spectra of **Calix-OCP-2-CBZ** (1.0×10^{-7} M in CH_3CN) upon addition of increasing amounts (2.5 - 72.6 equiv.) of $Cu(ClO_4)_2$ (λ_{exc} = 380 nm); (**b**) Binding isotherm for the fluorimetric titration of **Calix-OCP-2-CBZ** with Cu(II) with fitted curve and confidence intervals.

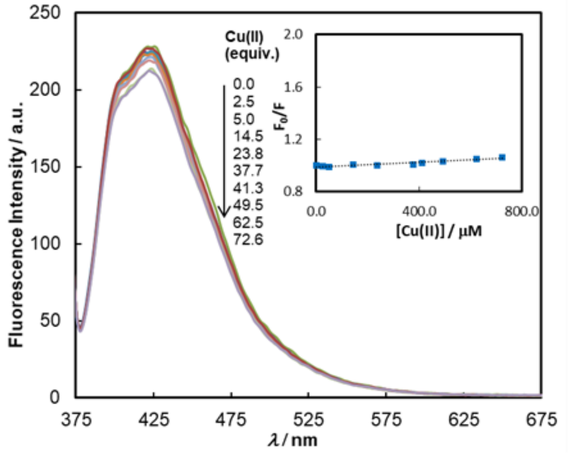

Figure 6. Emission spectra of **bis-Calix-TriPr-2-CBZ** (1.0×10^{-5} M in CH_3CN) upon addition of increasing amounts (2.5 - 72.6 equiv.) of $Cu(ClO_4)_2$ (λ_{exc} = 380 nm). Inset: Plot of F_0/F *vs* [Cu(II)].

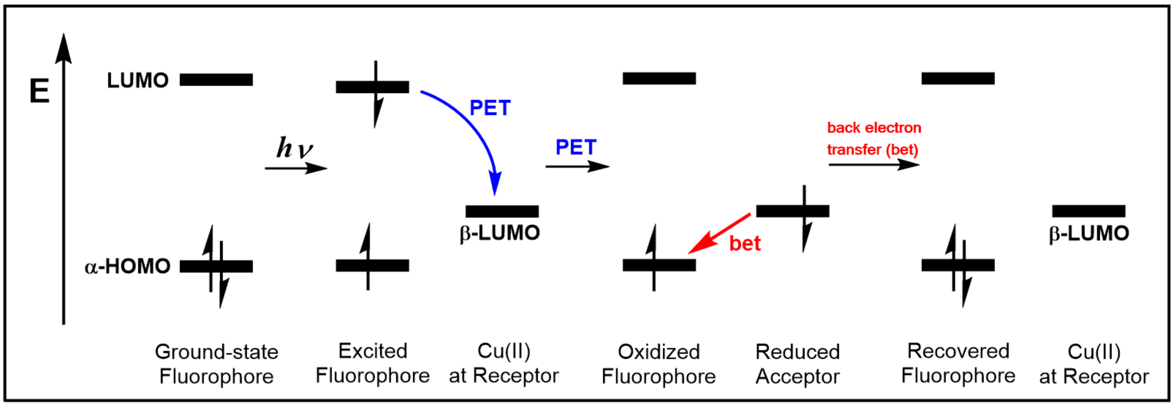

Figure 7. Cartoon illustrating the mechanism of fluorescence quenching by photoinduced electron transfer (PET).

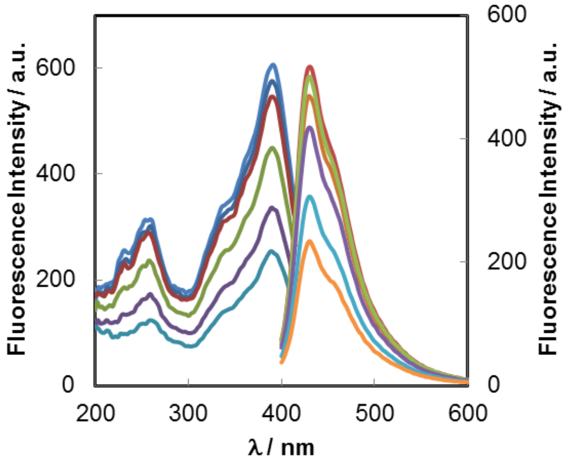

Figure 8. Excitation (left; monitored at 430 nm) and emission (right; λ_{exc} = 380 nm) spectra of polymer **5** (5.0×10^{-6} M in CH_3CN) upon addition of increasing amounts (0.25 - 10 equiv.) of $Cu(ClO_4)_2$.

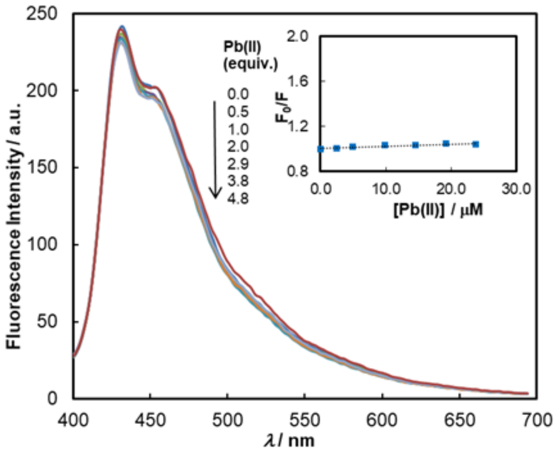

.

Figure S9. Emission spectra of polymer **5** (5.0×10^{-6} M in CH_3CN) upon addition of increasing amounts (0.5 - 4.8 equiv.) of $Pb(ClO_4)_2$ (λ_{exc} = 380 nm). Inset: Plot of F_0/F *vs* [Pb(II)].

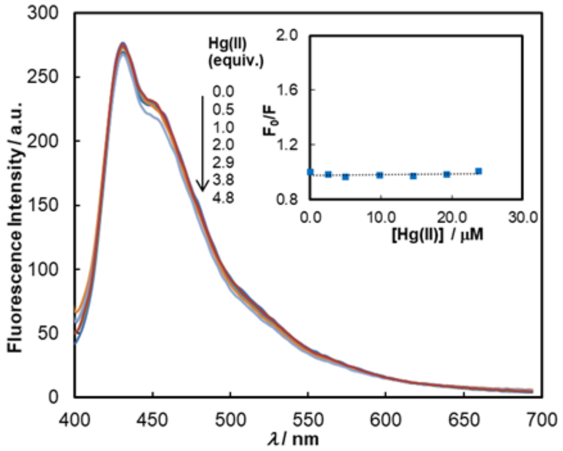

Figure 10. Emission spectra of polymer **5** (5.0×10^{-6} M in CH_3CN) upon addition of increasing amounts (0.5 - 4.8 equiv.) of $Hg(ClO_4)_2$ (λ_{exc} = 380 nm). Inset: Plot of F_0/F *vs* [Hg(II)].

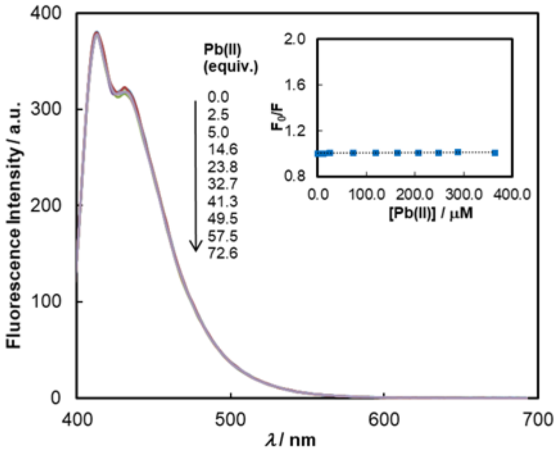

Figure S11. Emission spectra of **Calix-OCP-2-CBZ** (5.0×10^{-6} M in CH_3CN) upon addition of increasing amounts (2.5 - 72.6 equiv.) of $Pb(ClO_4)_2$ (λ_{exc} = 380 nm). Inset: Plot of F_0/F *vs* [Pb(II)].

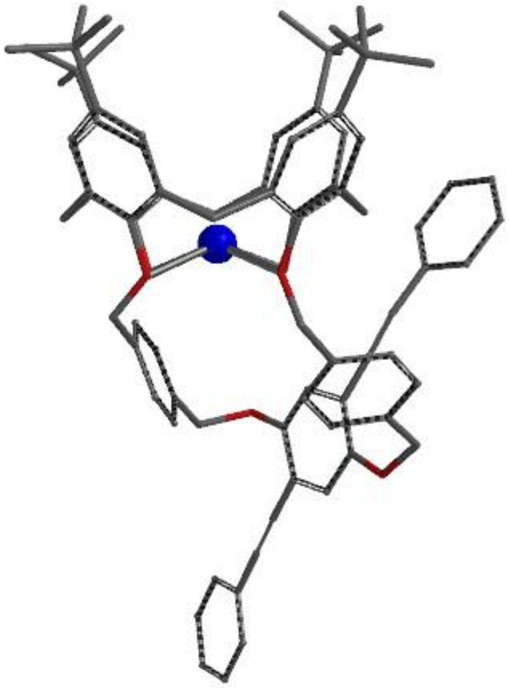

Figure 12. Optimised structure of Calix-OCP-Pb(II) complex (model). DFT calculations run at the B3LYP/6-31G(d) level of theory in vacuum, using the LANL2DZ pseudopotential for Pb [1]. Hydrogens omitted for clarity. Colour codes for elements: red = oxygen, grey = carbon, blue = lead.

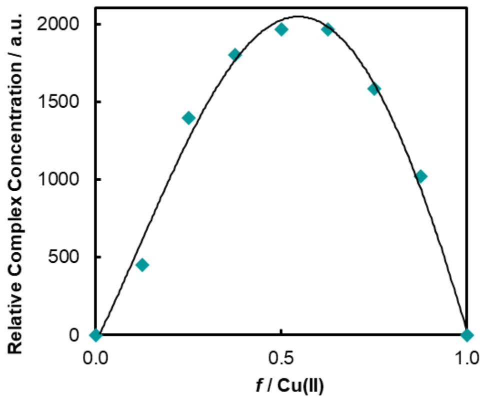

Figure 13. Job plot for complex formation between polymer **4** and Cu(II) in CH_3CN (at constant 1.0 x 10^{-5} M total concentration) as obtained from changes in fluorescence (λ_{exc} = 380 nm).

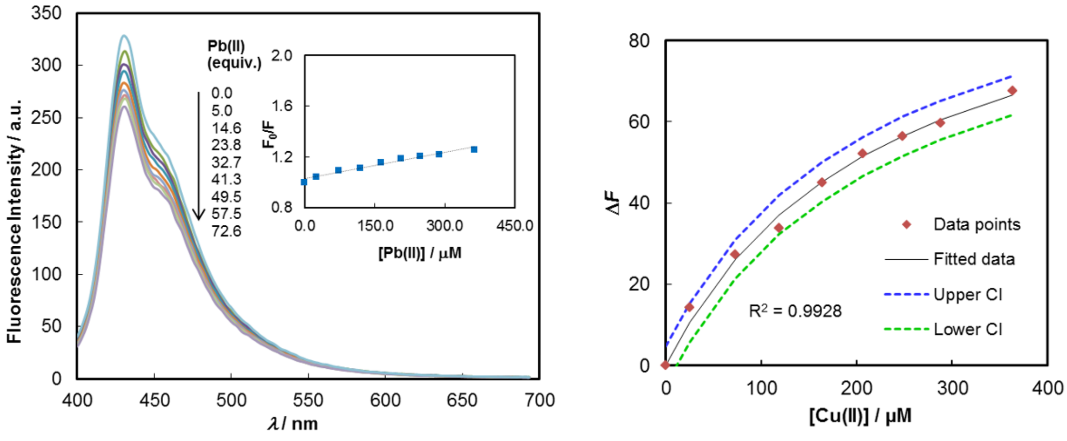

(**a**) (**b**)

Figure 14. (**a**) Emission spectra of polymer **4** (5.0 x 10^{-6} M in CH_3CN) upon addition of increasing amounts (5.0 - 72.6 equiv.) of $Pb(ClO_4)_2$ (λ_{exc} = 380 nm); (**b**) Binding isotherm for the fluorimetric titration of **4** with Pb(II) with fitted curve and confidence intervals.

Determination of stoichiometry of complexes from Job plots [2]

Table 1. Experimental values of $\Sigma c/c_{MAX}$ of the Job plot for polymer **5**.

nCu(II)/mol	x Cu(II)	F	[Polymer 5]/M	(F_0-F)/F_0 x [Polymer 5]/nM	c/c_{max}
0.00E+00	0.00	375.61	1.00E−05	0.00	0.00
2.50E−09	0.13	319.10	8.75E−06	1316.43	0.53
5.00E−09	0.25	265.05	7.50E−06	2207.71	0.89
7.50E−09	0.38	228.09	6.25E−06	2454.67	0.99
1.00E−08	0.50	188.58	5.00E−06	2489.68	1.00
1.25E−08	0.63	160.00	3.75E−06	2152.60	0.86
1.50E−08	0.75	112.83	2.50E−06	1749.05	0.70
1.75E−08	0.88	69.78	1.25E−06	1017.78	0.41
2.00E±08	1.00	18.17	0.00E+00	0.00	0.00
				**$\Sigma(c/c_{max})$ =**	**5.38**

x - molar fraction; F_0 is the fluorescent intensity of polymer **5** in the absence of copper cation; F is the fluorescent intensity recorded in the presence of metal cation; c is the concentration.

Table 2. Experimental values of Σc/cMAX of the Job plot for polymer **4**.

nCu(II)/mol	x Cu(II)	F	[Polymer 4]/M	$(F_0-F)/F_0$ x [Polymer 4]/nM	c/c_{max}
0.00E+00	0.00	450.90	1.00E−05	0.00	0.00
2.50E−09	0.13	427.24	8.75E−06	450.09	0.23
5.00E−09	0.25	365.43	7.50E−06	1393.65	0.71
7.50E−09	0.38	318.27	6.25E−06	1802.19	0.92
1.00E−08	0.50	270.05	5.00E−06	1965.93	1.00
1.25E−08	0.63	210.15	3.75E−06	1962.81	1.00
1.50E−08	0.75	159.45	2.50E−06	1584.11	0.81
1.75E−08	0.88	75.25	1.25E−06	1020.88	0.52
2.00E−08	1.00	2.23	0.00E+00	0.00	0.00
				**$\Sigma(c/c_{max})$ =**	**5.18**

x - molar fraction; F_0 is the fluorescent intensity of polymer **4** in the absence of copper cation; *F* is the fluorescent intensity recorded in the presence of metal cation; *c* is the concentration.

References

1. Spartan'14 Molecular Modeling Program, Wavefunction, Inc., Irvine, CA, 2014.
2. Olson, E.J.; Bühlmann, P. Getting More out of a Job Plot: Determination of Reactant to Product Stoichiometry in Cases of Displacement Reactions and n:n Complex Formation. *J. Org. Chem.* **2011**, 76, 8406–8412.
